# Disruption of *Trichoderma reesei cre2*, encoding an ubiquitin C-terminal hydrolase, results in increased cellulase activity

**DOI:** 10.1186/1472-6750-11-103

**Published:** 2011-11-09

**Authors:** Jai A Denton, Joan M Kelly

**Affiliations:** 1 School of Molecular and Biomedical Science, University of Adelaide, Adelaide, 5005, Australia

## Abstract

**Background:**

The filamentous fungus *Trichoderma reesei *(*Hypocrea jecorina) *is an important source of cellulases for use in the textile and alternative fuel industries. To fully understand the regulation of cellulase production in *T. reesei*, the role of a gene known to be involved in carbon regulation in *Aspergillus nidulans*, but unstudied in *T. reesei*, was investigated.

**Results:**

The *T. reesei *orthologue of the *A. nidulans creB *gene, designated *cre2*, was identified and shown to be functional through heterologous complementation of a *creB *mutation in *A. nidulans*. A *T. reesei *strain was constructed using gene disruption techniques that contained a disrupted *cre2 *gene. This strain, JKTR2-6, exhibited phenotypes similar to the *A. nidulans creB *mutant strain both in carbon catabolite repressing, and in carbon catabolite derepressing conditions. Importantly, the disruption also led to elevated cellulase levels.

**Conclusions:**

These results demonstrate that *cre2 *is involved in cellulase expression. Since the disruption of *cre2 *increases the amount of cellulase activity, without severe morphological affects, targeting *creB *orthologues for disruption in other industrially useful filamentous fungi, such as *Aspergillus oryzae*, *Trichoderma harzianum *or *Aspergillus niger *may also lead to elevated hydrolytic enzyme activity in these species.

## Background

Environmental sustainability and fossil fuel supply concerns have caused increased focus and research activity in the area of alternative fuel production. The filamentous fungus *Trichoderma reesei *(*Hypocrea jecorina*) has been used for cellulase production over many decades, and the release of the complete genome sequence facilitates studies that will extend our understanding of how cellulases are regulated in this organism. This understanding may present further opportunities for targeted gene manipulation to increase cellulolytic enzyme production. In the presence of glucose and other easily metabolised carbon sources, genes encoding cellulase enzymes are not highly expressed, even when inducer is present, due to carbon catabolite repression [Reviewed [1,2]]. An industrially important cellulolytic strain of *T. reesei*, Rut-C30, was selected for improved cellulase production following multiple rounds of mutagenesis and selection. The Rut-C30 genome contains a mutation in the gene encoding a transcriptional repressor involved in carbon catabolite repression, *cre1 *[3]. Many other mutations have also been identified in Rut-C30 [4-6], and those analysed after identification by array comparative genomic hybridization, including an 85 kb deletion containing 29 genes, do not affect cellulase production [6]. These studies show that mutagenesis and selection lead to many off target genetic changes, and some of these may be undesired. A complete understanding of the complex regulation of cellulose metabolism may open up the opportunity for precise targeted genetic manipulation of *T. reesei *strains for increased cellulase production.

Genetic analysis in the model filamentous fungus, *Aspergillus nidulans*, has provided a framework for the study of the mechanism of carbon repression in *T. reesei*. In *A. nidulans *there are three genes in which mutations have been identified resulting in partial deregulation of carbon repression: *creA*, the *A. nidulans *orthologue of *cre1*, encoding a zinc finger DNA binding transcriptional repressor [7,8]; *creB*, encoding a ubiquitin C-terminal hydrolase [9]; and *creC*, encoding a WD40 repeat protein shown to be present in a complex with CreB [10]. Orthologues of *creA *have been identified and studied in numerous fungi including *A. niger *[11], *Humicola grisea *[12], *Cochliobolus carbonum *[13], *Gibberella fujikuroi *[14] and *Botrytis cinerea *[14], however *creB *and *creC *orthologues remain largely unstudied in other fungi. Mutations in the *creB *and *creC *genes lead to partial deregulation of carbon repression of some genes, and affect the expression of other genes, but they do not cause the severe morphological impairment caused by *creA *mutations [15]. In *A. nidulans *there is evidence that CreA is a direct target of CreB suggesting that CreB functions in carbon repression via CreA [16]. Disruption of carbon source mediated repression without severe morphological impairment could potentially lead to the development of industrially useful fungal strains, and as such *creB *represents a candidate for targeted disruption.

The deletion of *cre1 *within the progenitor strain of *T. reesei*, QM 6a, has recently been characterised [17]. As with *A. nidulans *strains containing *creA *mutations, the deletion of *cre1 *in this non mutated background resulted in higher total secreted protein and enzymatic activity of endoglucanases and xylanases, as well as morphological impairment. Cellulase regulation in *T. reesei *has also been shown to involve transcriptional regulators including the transcriptional repressor Ace1 [18,19], and the transcriptional activators and Ace2 [20] and Xyr1 [21,22]. It has recently been shown that *xyr1 *transcription is repressed by Cre1, and it was proposed that Ace1 might also play a role in *xyr1 *repression [23]. Deletion of *xyr1 *in *T. reesei *resulted in loss of transcription of the major cellulase encoding genes, *cbh1*, *cbh2 *and *egl1 *[22], and furthermore there was no secreted cellulase or xylanase activity detected in the *xyr1 *deletion strain after 72 hours growth in inducing conditions [22]. Xyr1 has also been implicated in the expression of Egl3 [24] and the induction of the lactose metabolism pathway by lactose [25].

We find no sequence similar to *ace2 *in the *A. nidulans *genome, highlighting the differences between regulation of the cellulases in the two filamentous fungi. In this study we have shown bioinformatically that the *T. reesei *genome contains orthologues of the *A. nidulans creB *and *creC *genes. There have been no reports of mutations in *creB *and *creC *orthologues in *T. reesei*, and it was possible that mutations in the *T. reesei *orthologues of *creB *or *creC *may not result in the equivalent phenotypes seen in *A. nidulans*, as regulation of cellulase encoding genes in *T. reesei *could have been independent of the CreB/CreC complex. We have cloned the *cre2 *gene and shown that it is a functional orthologue of the *A. nidulans creB *gene. A *T. reesei *strain containing a disruption within the *cre2 *encoding region was generated and the phenotypic effects analysed.

## Results

### The *T. reesei *Genome Contains Homologues of *creB *and *creC*

**Figure 1:**
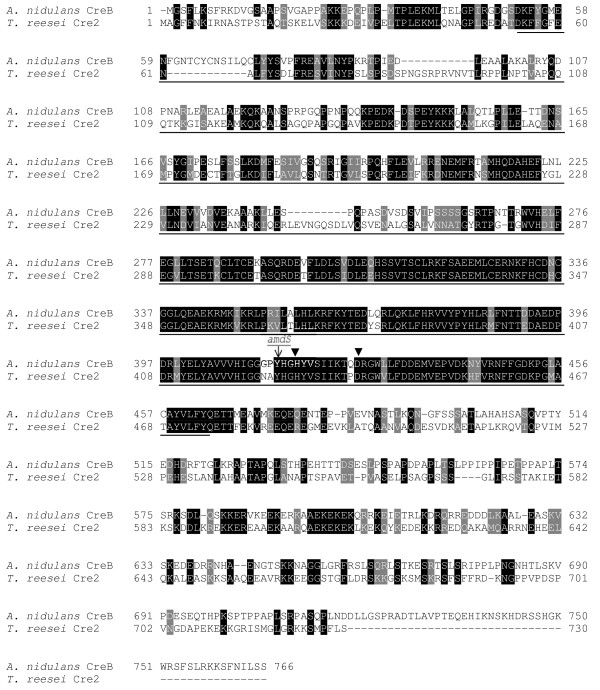
**Protein sequence alignment of *A. nidulans *CreB and the putative *T. reesei *orthologue**. Black box denotes identical amino acids. Grey box denotes similar amino acids. Solid underline denotes the ubiquitin hydrolayse domain, Asp^54 ^to Tyr^474^. The position of insertion of the *A. nidulans amdS *region is indicated, between Tyr^425 ^and His^426^. Arrows denote the active sites, His^428 ^and Asp^437 ^of the ubiquitin hydrolase domain [26].

The *T. reesei *genome was examined to identify potential orthologues of the *A. nidulans creB *and *creC *genes. Using protein sequences for CreB (AAL04454) and CreC (AAF63188) from the National Centre for Biotechnology Information website http://www.ncbi.nlm.nih.gov, TBLASTN analyses were performed against the *T. reesei *translated nucleotide database http://genome.jgi-psf.org/Trire2/Trire2.home.html. Both the identified genomic regions and deduced protein sequences of the *T. reesei *CreB and CreC orthologues, designated Cre2 (Protein ID 122405) and Cre3 (Protein ID 64608) were aligned to the *A. nidulans *orthologues using the Geneious software package (Biomatters Ltd, USA). The Cre2 amino acid sequence showed 43% identity to *A. nidulans *CreB (Figure 1), while the Cre3 sequence showed 48% identity to *A. nidulans *CreC.

### Cre2 is a Functional Orthologue of CreB

**Figure 2:**
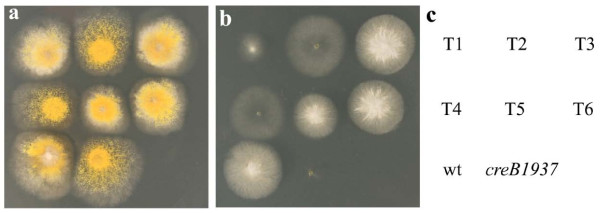
**Complementation of *A. nidulans creB1937 *by *T. reesei cre2***. *The A. nidulans creB1937 *mutant strain was co-transformed with pPL3 and pTRcre2 containing *Trichoderma reesei cre2*, and six riboflavine independent transformants, T1-T6, were tested for complementation of *creB1937 *phenotypes. Strains were incubated at 37°C on a) 1% glucose, 10 mM ammonium tartrate; b) 1% glucose, 10 mM ammonium tartrate, 10 mM allyl alcohol; c) key to strains. Plates were supplemented with riboflavin and para amino benzoic acid.

An *A. nidulans *strain that contained the *riboB2 *and *creB1937 *mutations was transformed with pPL3 containing *riboB^+^*, with pTRcre2, containing *T. reesei cre2 *also present in the transformation mix. The *creB1937 *mutation leads to a pleiotropic phenotype, including sensitivity to allyl alcohol in the presence of glucose and poor utilisation of proline. Six riboflavin independent transformants were tested for co-transformation of pTRcre2 via complementation of these *creB1937 *phenotypes (Figure 2). Transformants T3 and T6 were morphologically similar to wildtype (Figure 2a), and also showed complementation of the phenotypes caused by *creB1937 *of sensitivity to allyl alcohol in the presence of glucose (Figure 2b), and poor proline utilisation. T1, T2, T4 and T5 showed varying degrees of partial complementation, possibly due to differences in expression levels due to copy number or position of integration affects, but these were not studied further as the aim was to show that Cre2 had the same function as CreB, rather than a complete analysis of the transformants. Thus the *T. reesei cre2 *gene is a functional orthologue of *creB*, and *cre2 *can be expressed in *A. nidulans *from the *T. reesei cre2 *promoter.

### Generation of a *cre2 *Disruption Strain

A plasmid, pTRcre2ΔamdS, was constructed to disrupt *cre2 *in *T. reesei*, in which the *A. nidulans amdS *gene containing the *amdSI9 *promoter mutation was inserted into the *cre2 *ORF disrupting the gene within the ubiquitin C-terminal hydrolase domain. The position of the insertion of the *A. nidulans amdS *encoding region is indicated in Figure 1, between Tyr^425 ^and His^426^, which is prior to the essential active sites of the ubiquitin hydrolase domain, His^428 ^and Asp^437 ^[26], and thus this insertion will create a non-active allele. A strain containing a disruption of the endogenous *cre2 *gene, JKTR2-6, was generated by transformation of QM 6a with this plasmid. After purification, homologous recombination at the *cre2 *locus, required for disruption of the ORF, was verified using Southern analysis.

### Meiotic Segregation of the Disruption Phenotype with the *amdI9 *Phenotype

With the recent discovery of strains of opposite mating type [27], meiotic crossing can be performed in *T. reesei*. The disruption construct contains the *amdI9 *selection marker, and this can be followed in a genetic cross through scoring the strong growth on acrylamide. To provide evidence that the phenotypes observed were due to the disruption and not a fortuitous change elsewhere in the genome, JKTR2-6 was crossed to CBS999.97, a strain shown to undergo sexual reproduction with QM 6a [27]. The pleiotropic phenotype attributed to *cre2 *disruption always segregated with the *amdI9 *phenotype in the progeny of a sexual cross. Of 38 progeny screened, 23 grew on acrylamide due to the *amdI9 *marker and all 23 also showed the pleiotropic phenotype. This segregation of the *cre2 *disruption phenotype with the *amdI9 *marker shows that the pleiotropic phenotype seen in the disrupted strain segregates together and is not due to fortuitous changes elsewhere in the genome.

### Phenotypic Effects of *cre2 *Disruption on mycelial growth

**Figure 3:**
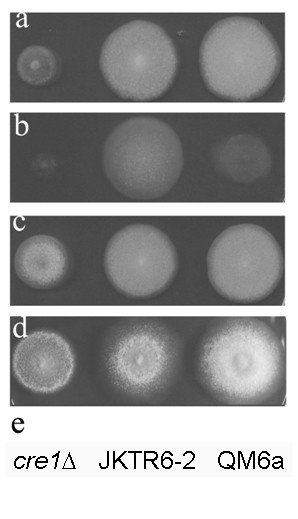
**Phenotype of *T. reesei cre2 *disruption strain on solid media**. Strains were grown at 30°C on a) 1% glucose, 10 mM ammonium tartrate; b) 1% maltose, 10 mM ammonium tartrate; c) 1% cellobiose, 10 mM ammonium tartrate; d) 1% glucose, 10 mM proline; e) Key to strains. 0.01% triton-X was added to all plates for photography, but plates without Triton-X showed essentially identical results.

JKTR2-6 showed similar mycelial growth but slightly reduced conidiation compared to QM 6a on solid minimal media with glucose as the carbon source, and considerably stronger mycelial growth and conidiation compared to the *cre1 *deletion strain on this medium (Figure 3a). JKTR2-6 grew much better than QM 6a on solid medium containing maltose (Figure 3b), a phenotype linked to increased α-glucosidase activity that has also been shown for *A. nidulans *strains containing *creB *mutations [15], whereas the *T. reesei cre1 *deletion strain grew very poorly on this medium. QM 6a and JKTR2-6 grew similarly when cellobiose was the sole carbon source, suggesting limited effects on β-glucosidase activity (Figure 3c). JKTR2-6 showed poorer growth than QM 6a or the *cre1 *deletion strain on medium containing proline as the sole nitrogen source (Figure 3d), a phenotype also seen with *A. nidulans creB *mutant strains. However, unlike *creB *mutations in *A. nidulans *which also grow poorly on lactose, JKTR2-6 had no discernable effect on lactose utilization in solid medium (data not shown) but effects were apparent in liquid conditions (see below). JKTR2-6 also had higher protease activity than QM 6a on medium containing 5% (vol/vol) liquid soy milk, as assessed by the size of the cleared zone around the colonies, but not when 1% glucose was also present in the medium.

**Figure 4:**
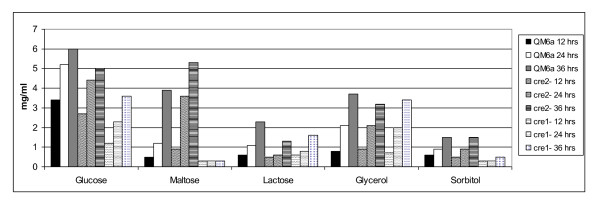
**Total Dry Biomass in Various Carbon Sources**. Biomass was harvested from QM 6a (+), JKTR2-6 (*cre2^-^*) and the *cre1 *deletion strain (*cre1^-^*) after growth on the carbon source indicated for 12, 24 and 48 hours at 30°C. The biomass was dried and weighed, and the dry weights are shown in mg per ml, averaged across three replicates. Data and statistical significance are shown in Additional File 1.

Growth tests were also performed in 50ml shake flask culture. Dry biomass weights were determined for a range of carbon sources for each of the three strains (Figure 4; Additional File 1). JKTR2-6 had significantly (P < 0.05) less biomass than QM 6a on glucose at 24 and 36 hours, but significantly (P < 0.001) more than the *cre1 *disruption. Solid media growth tests indicated greatly improved growth on maltose for JKTR2-6 and extremely poor growth for the *cre1 *disruption, which was corroborated in the liquid culture experiments. JKTR2-6 had three times more biomass than QM 6a at 24 hours post inoculation while the *cre1 *disruption failed to grow. Analysis of liquid cultures showed that disrupting *cre2 *reduced growth on lactose relative to QM 6a, a result that reflects the phenotype of *creB *mutations in *A. nidulans *but that was not detectable for the *T. reesei *mutation on solid medium. The disruption of *cre1 *or *cre2 *had no noticeable effect in medium with 2% glycerol as the sole carbon source, and both strains had similar amounts of biomass compared to QM 6a. The growth of JKTR2-6 on 2% sorbitol was very similar to QM 6a, while the *cre1 *disruption grew very poorly.

### Effects of *cre2 *Disruption on cellulase activity

#### Sophorose as inducer

In a preliminary experiment, total secreted cellulase activity was assayed in 5 ml shake cultures in medium containing glucose as a repressing carbon source and sophorose as an inducer. When glucose was present, JKTR2-6 and the *cre1 *deletion strain showed high levels of secreted cellulase activity after 6, 12 and 18 hours, whereas QM 6a had limited cellulase activity after 6 and 12 hours, but the activity had increased to levels similar to the disruption strains by 18 hours. Glucose levels were determined in QM 6a culture media using HPLC, and were 0.03% at 12 hours and 0% at 18 hours, and thus by 18 hours the cultures contained no source of repression. When sorbitol, a nonrepressing carbon source, replaced glucose, JKTR2-6 showed higher cellulase activity than either QM 6a or the *cre1 *deletion strain at all time points (Additional File 2). These experiments indicated that *cre2 *had an effect on cellulase expression, but the small culture volumes (due to sophorose cost), absence of accurate mycelia mass, and complications in dissecting effects on repression from those on uptake of sophorose [28] made interpretation difficult. Based on these preliminary observations, we undertook further experiments in larger volumes using two inducers, lactose, the catabolism of which is initiated by extracellular hydrolysis, thus reducing the effects of inducer exclusion, and microcrystalline cellulose (Avicel).

#### Lactose as inducer

**Figure 5:**
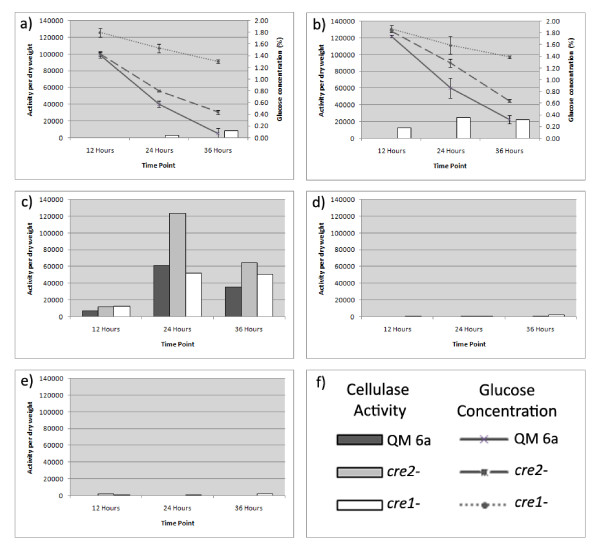
**Total secreted Cellulase in Lactose induced cultures**. Cellulase secretion per gram dry weight in *T. reesei *strains QM 6a, JKTR2-6 and *cre1 *deletion, 12, 24 and 36 hours post transfer. Mycelia for inoculation were harvested after growth for 24 hours in 1% (w/v) glucose medium. Mycelia were washed with liquid carbon free medium and 200 mg was added to 50 ml culture medium in 250 ml Erlenmeyer flasks. Cultures were grown at 30°C, shaken at 200 RPM, and were harvested 12 hours, 24 hours and 36 hours post inoculation. Total secreted cellulase activity was measured using the EnzChek Cellulase Substrate expressed as fluorescence per mg. Dry weights are shown in Additional File 1 and levels of total cellulase activity are shown in Additional File 3. Glucose concentrations were determined by HPLC analysis. The carbon sources for each of the cultures are a) 2% glucose, b) 2% glucose and 2% lactose, c) 2% lactose, d) 2% sorbitol and e) 2% glycerol. The strain key is f). The data presented is based on biological triplicates that were assayed in duplicate.

In experiments using lactose as the inducer, strains were grown in 50 ml shake cultures, which allowed accurate measurement of mycelial dry weights to determine activity per gram dry weight. There was little cellulase activity detected in any of the strains tested in glucose (Figure 5a), sorbitol (Figure 5d) or glycerol (Figure 5e) media without added inducer. In medium containing both glucose and lactose as inducer, for QM 6a, cellulase activity was not detected as the cellulase encoding genes are subject to glucose repression. In the *cre1 *deletion strain, cellulase activity was detected consistent with previous work indicating that at least one major exocellulase was not subject to carbon repression in the *cre1 *deletion strain (Figure 5b). In JKTR2-6 there was no detectable cellulase activity until the 36 hour time point in 2% lactose and glucose indicating very modest levels of derepression (Additional File 3). After 36 hours glucose concentrations in the cultures had dropped to 0.3% for QM 6a and 0.6% for JKTR2-6, but no activity could be detected in the QM 6a cultures. In medium containing lactose as an inducer, but lacking a repressing carbon source, cellulase activity per gram dry weight was higher in JKTR2-6 compared to QM 6a across all three time points, particularly at 24 hours (Figure 5c).

Total secreted xylanase activity was also measured in 2% glucose, 2% glucose/2% lactose and 2% lactose cultures. As with the cellulase assays, JKTR2-6 showed elevated total activity by weight when compared to QM 6a on 2% lactose (Additional File 3).

#### Microcrystalline cellulose as inducer

**Figure 6:**
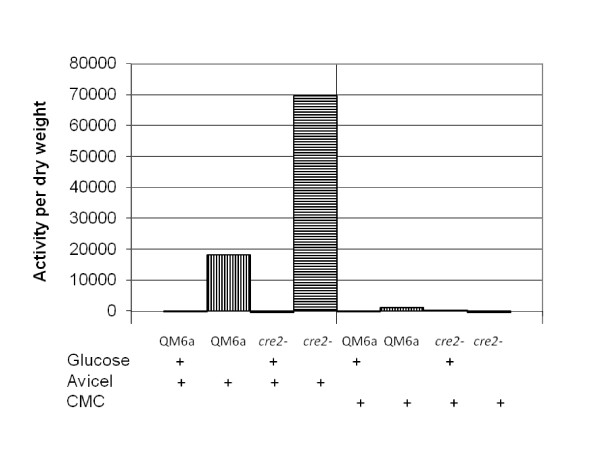
**Total secreted cellulase in Avicel induced cultures**. Cellulase secretion in *T. reesei *strains QM 6a and JKTR2-6 grown for 24 hours post transfer. Conidia were added to 100 ml culture medium in 250 ml Erlenmeyer flasks. Cultures were grown at 30°C, shaken at 1500 RPM. Total secreted cellulase activity was measured using the EnzChek Cellulase Substrate expressed as fluorescence/mg dry weight. Dry weights are shown in Additional File 4. The carbon sources shown are 1% glucose plus 0.5% avicel, 0.1% fructose plus 0.5% avicel, 1% glucose plus 0.5% carboxymethyl cellulose, and 0.1% fructose plus 0.5% carboxymethyl cellulose. No cellulase was detected using this detection system in either strain or condition using CMC as inducer. The data presented is based on biological triplicates that were assayed in duplicate.

Since elevated expression was observed with lactose as an inducer after 24 hours induction, we also tested this time point using Avicel and carboxy methyl cellulose as inducers. We grew 100 ml cultures of QM 6a and JKTR2-6 for 24 hours in media containing 0.5% avicel, with or without 1% glucose. In both QM 6a and JKTR2-6 there was no detectable cellulase activity in any of the cultures containing glucose. In the cultures containing inducer without repressor, there was significantly more cellulase activity per gram dry-weight mycelium in JKTR2-6 compared to QM 6a (Figure 6). We also grew 100 ml cultures of QM 6a and JKTR2-6 for 24 hours in media containing 0.5% carboxymethyl cellulose, with or without 1% glucose, but in these culture conditions, carboxymethyl cellulose did not induce any cellulase that was detectable using the ENZ-CHEK cellulase assay substrate as the detection system. Because carboxy methyl cellulase has been detected in these conditions previously, we assume that this is due to a limitation of the detection substrate.

## Discussion

The *T. reesei *Cre2 amino acid sequence is conserved with *A. nidulans *CreB, particularly within the region encoding the ubiquitin C-terminal hydrolase domain, and complementation of the pleiotropic phenotype of the *A. nidulans creB1937 *mutant by the *T. reesei cre2 *gene showed it is a functional orthologue of *creB*. The effects of CreB on carbon catabolite repression and the effects on the regulation of permeases are proposed to operate via separate mechanisms, and thus the complementation of the range of phenotypes indicates that both functions are conserved between the two orthologues.

While it is not uncommon for filamentous fungal genes expressed from their endogenous promoters to function in other fungal species, such as the *amdS *gene encoding acetamidase [29], CreB forms part of a large complex with CreC [10], and is likely to require multiple protein-protein interactions for its functions. Therefore the complementation of the *creB1937 *mutation by *cre2 *is of importance as it demonstrates that the CreB/CreC complex previously identified in *A. nidulans*, and also the targets of CreB deubiquination, are conserved between distant filamentous fungi despite relatively low conservation outside of the ubiquitin C-terminal hydrolase domain.

We made a *cre2 *disruptant strain using molecular genetic techniques, and the phenotype of the disruptant is consistent with published phenotypes of *creB *mutations in *A. nidulans*. We used both Southern analysis and meiotic crossing to show that the *cre2 *disruption was the result of a double cross over event at the *cre2 *locus, and that the phenotype was due to the *cre2 *disruption and not to a fortuitous alteration to the genome that occurred in the transformation. The discovery of a sexual cycle in *T. reesei *[27] allows for the first time traditional genetic analysis of industrial strains, allowing alternatives to molecular approaches. In this case, a meiotic cross was used to demonstrate that the selectable marker phenotype and the *cre2 *disruption phenotype co-segregate, thus strengthening that the disruption phenotype was due to the *cre2 *disruption and not to a fortuitous alteration to the genome.

Growth testing on both solid and liquid media revealed similar phenotypes between *T. reesei *and *A. nidulans *strains containing *cre2*/*creB *mutations. These growth tests support the initial hypothesis that a strain with disrupted *cre2 *will lack the extreme morphological impairment of *cre1 *mutants, although the disruption of *cre2 *does lead to somewhat impaired growth on glucose and lactose, evident in liquid culture.

In the absence of glucose, disruption of *cre2 *leads to elevated cellulase activity whether sophorose, lactose or microcrystalline cellulose was used as an inducer, but not when a source of induction was absent, and this elevated cellulase activity in induced conditions is a robust phenotype of the *cre2 *disruption. When glucose is also present with an inducer, disruption of *cre2 *leads to only a very slight relaxation of glucose repression compared to QM 6a, however this derepression is much less than that due to disruption of *cre1*.

A full description of the functional roles of CreB has yet to be determined, even in *A. nidulans*. The CreB protein has been shown to be a functional deubiquitinase in an *E. coli *assay (9), and its direct effect on permeases have been shown for the quinate permease (16; Kamlangdee and Kelly, unpublished), and effects on permeases are likely to account for the reduced growth on some sole carbon sources. In addition to these effects on growth in various carbon sources, the *A. nidulans creB *mutations also lead to carbon catabolite derepression of a number of enzyme activities in the presence of a source of repression, including alcohol dehydrogenase induction by ethanol which requires no permease. Thus the full range of pleiotropic phenotypes are unlikely to be accounted for solely by effects on permeases. The range of phenotypes due to lack of function mutations in *A. nidulans *(9), *Aspergillus oryzae *(Hunter and Kelly, unpublished) and *T. reesei *orthologues are broadly similar, and thus the functions are likely to be conserved across these organisms. The effects on the induction of activities of enzymes found to be elevated in mutations in *A. nidulans *and *A. oryzae *involve effects on transcription, and preliminary results indicate that this is likely also to be the case in *T. reesei *(Morris, Hunter and Kelly, unpublished). Thus it is likely that CreB plays a role in transcriptional regulation, as well as its effects on the stability of permeases.

Since the disruption of *cre2 *increases the amount of cellulase activity without the severe morphological deficiencies seen with the *cre1 *disruption, targeting *creB *orthologues for disruption in other industrially useful filamentous fungi, such as *A. oryzae*, *Trichoderma harzianum *or *A. niger *may also prove beneficial. While the disruption of *cre2 *has increased secreted cellulase activity, further improvements could potentially be made using JKTR2-6 as a foundation for further targeted genetic manipulation. Examples of potential manipulations include making a *cre1 cre2 *double null strain, and the over expression of the *T. reesei *orthologue of *creD *[30], shown in *A. nidulans *to increase derepression in a *creB *null background (R. Lockington, personal communication). Protease encoding genes have previously been targeted for disruption to improve heterologous protein expression in both *A. oryzae *[31,32] and *A. niger *[33]. *T. reesei *proteases could also be targeted in these strains to potentially improve extracellular cellulase activity.

## Conclusions

We have identified and disrupted the *cre2 *gene in *T. reesei*, and shown that it is the functional homologue of the *creB *gene in *A. nidulans*. The disrupted strain shows a similar phenotype to the equivalent *A. nidulans *mutant. We have shown that the disruption of *cre2 *increases the amount of cellulase activity in the presence of an inducer, without severe morphological affects. Therefore, we propose that targeting *creB *orthologues for disruption in other industrially useful filamentous fungi, such as *A. oryzae*, *T. harzianum *or *A. niger *may also lead to elevated hydrolytic enzyme activity in these species, and we are presently investigating these possibilities.

## Methods

### Strains

The *A. nidulans *strains used were *creB1937 *(*yA1 pabaA1; creB1937; riboB2*) and wild-type (*yA1 pabaA1; riboB2*). The *T. reesei *strains used were QM 6a (wild type), CBS999.97 (containing MAT1-1, (27)), VTT-D 02877 (containing *cre1::amdS*, [17]) and JKTR2-6 (containing *cre2::amdS*, this study). *Escherichia coli *strain DH5α (*supE44 ΔlacU169 *(*Φ80 lacZ ΔM15*) *hsdR17 recA1 endA1 gyrA96 thi-1 relA1*) was used to propagate plasmids.

### Media

Solid and 5 ml liquid fungal media were based on that described by Cove [34] or potato dextrose agar (PDA) where stated, while 50 ml and 100 ml liquid medium was as described by Seiboth *et al*. [35]. *A. nidulans *and *T. reesei *were grown at 37°C and 30°C respectively. Nitrogen sources were added to a final concentration of 10 mM and carbon sources at 1% (wt/vol), unless otherwise stated. Riboflavine and para amino benzoic acid were added to media at final concentrations of 2.5 μg per ml and 0.5 μg per ml, respectively, when required. All *T. reesei *growth tests were initially conducted on detergent free solid media but for photography, TritonX-100 was added to *T. reesei *media at 0.01% (vol/vol) to reduce colony diameter.

### *T. reesei *Sexual Cross

The *T. reesei *sexual cross was performed as described in Seidl *et al*. [27] between JKTR2-6 (*cre2::amdS*) and CBS999.97 (MAT1-1) on PDA at 25°C with 16 hours of light in a 24 hour period. Fruiting bodies formed approximately 16 days post inoculation and spores were collected from the lid of the petri dish on day 22.

### Transformation

*A. nidulans *was transformed based on the method described by Tilburn *et al*. [36], and transformants were selected for riboflavine independence conferred by the *riboB^+ ^*gene in pPL3 [37]. *T. reesei *was transformed using linearised plasmid based on the method described by Pentillä *et. al *[38]. Transformants were selected on medium containing 10 mM acetamide as the sole nitrogen source using the *amdI9 *variant of the *amdS *gene from *A. nidulans*. This *amdI9 *variant leads to higher expression of acetamidase, and thus easier selection of single copy transformants. Bacteria were transformed as described in Sambrook and Russell [39].

### Molecular methods

Molecular methods were as described by Sambrook and Russell [39]. Bacterial plasmids were purified using the Wizard Plus SV Minipreps DNA Purification System (Promega, USA). Fungal DNA was extracted using the DNeasy Plant Mini Kit DNA purification system (Qiagen, USA). All PCRs were performed using the high fidelity polymerase, Phusion (Genesearch, Australia). Southern analysis was performed using DIG Highprime Labelling and Detection Kit (Roche, Australia).

### Generation of *cre2 *Constructs

A 4505 nucleotide region encompassing the Cre2 coding region, including approximately 500 bp upstream of the putative start codon and approximately 200 bp downstream of the putative stop codon, was amplified using primers CBUP1 (5'CCCATTGCTGTCTCGCTATT3') and CBDOWN2 (5'AAGGCAAGATGTGTCGGAAC3'). The amplicon was cloned into pBluescript generating pTRcre2 for use in heterologous complementation analysis. The disruption construct, pTRcre2ΔamdS, was generated through ligation of the *A. nidulans amdS *encoding region, containing the *amdI9 *promoter mutation, into a MscI restriction site. This restriction site occurs prior to the codon for His426, which is within the 300 amino acid ubiquitin hydrolase domain and prior to His428 and Asp437 shown to be active sites [26].

### Analytical Methods

Mycelia from fungal strains QM 6a, VTT-D 02877, the *cre1 *disruption, and JKTR2-6, the *cre2 *disruption, were cultured either in small 5 ml cultures in *A. nidulans *medium described by Cove [34], on in larger 50 ml or 100 ml cultures in *T. reesei *medium described by Seiboth *et al*. [35].

Total cellulase or xylanase enzyme activity was assayed either using the ENZ-CHEK cellulase assay substrate or the ENZ-CHEK xylanase assay kit (Invitrogen, USA) following the manufacturer's instructions, using a Molecular Devices SpectraMax Plus384 Absorbance Microplate Reader. These assays determine the relative cellulase or xylanase activity between strains within a single experiment, by the measurement of fluorescence at 360/460 nm produced by cellulase or xylanase activity on the substrates. The manufacturer's standards indicate that a fluorescence of 40000 corresponds to approximately 6 mU/mL of cellulase activity for the cellulase substrate, while a fluorescence of 1000 corresponds to approximately 450 mU/mL of xylanase activity using the xylanase kit.

The biomass from each culture was harvested, dried at 65°C and weighed.

A Rezex ROA-Organic analysis column (300 × 7.8 mM, Phenomenez, Australia) and a refractive index detector (Model 350, Varian, Australia) were used to analyse glucose concentrations. The mobile phase was ultra pure water at a flow rate of 0.6 ml/min and the column was maintained at 35°C.

## Authors' contributions

JD carried out project design, research work and drafting of the manuscript. JK was responsible for supervision, initial project conception and editing of the manuscript. All authors read and approved the final manuscript.

## Supplementary Material

Additional file 1 **Total Dry Biomass in Various Carbon Sources**. Biomass was harvested from strains grown on carbon sources as shown for 12, 24 and 36 hours at 30°C. The strains are QM 6a, JKTR2-6, and the *cre1 *deletion strain. Dry weight is shown in mg per ml. Click here for file

Additional file 2 **Total secreted cellulase activity of three *T. reesei *strains**. Cellulase secretion of *T. reesei *strains QM 6a (dark grey shading), JKTR2-6 (light grey shading) and *cre1 *deletion (white shading) measured using the EnzChek Cellulase Substrate. Mycelia for inoculation were harvested after growth for 28 hours in 1% (w/v) glucose medium. Mycelia were washed with liquid carbon free medium and 50 mg was added to 5 ml culture medium in 10 ml culture bottles. Cultures were grown at 30°C, shaken at 200 RPM, and were harvested 6 hours, 12 hours and 18 hours post inoculation. Time indicated as hours post transfer. Error bars indicate standard deviations of duplicate cultures each analysed in duplicate. (a) Repressing conditions, growth in medium containing 1% glucose and 1 mM sophorose. (b) Derepressing conditions, growth in medium containing 1% sorbitol and 1 mM sophorose. When diluted, the measured cellulase activity of these samples decreased proportionally, showing the detection limit of the assay had not been exceeded. Click here for file

Additional file 3 **Secreted Cellulase and Xylanase Activities**. Cellulase (a) or Xylanase (b) secretion in *T. reesei *strains QM 6a, JKTR2-6, and the *cre1 *disruption strain measured using the EnzChek Cellulase Substrate (a) or the EnzChek Xylanase Assay Kit (b) uncorrected for dry weight. Time indicated as hours post transfer, grown at 30°C. Error margins indicated standard deviations determined from the result of assay duplicates and biological triplicates. Click here for file

Additional file 4 **Dry weights for Avicel and Carboxymethyl Cellulose cultures**. Data for Figure 6. Conidia were added to 100 ml culture medium in 250 ml Erlenmeyer flasks. Cultures were grown at 30°C, shaken at 1500 RPM, for 24 hours, in triplicate. The carbon sources are 1% glucose plus 0.5% avicel, 0.1% fructose plus 0.5% avicel, 1% glucose plus 0.5% carboxymethyl cellulose, 0.1% fructose plus 0.5% carboxymethyl cellulose. Click here for file
