# Supplementary material for: Disruption of Trichoderma reesei cre2, encoding an ubiquitin C-terminal hydrolase, results in increased cellulase activity
Source: BMC Biotechnol. 2011 Nov 9;11:103. doi: 10.1186/1472-6750-11-103 (PMC3226525; doi:10.1186/1472-6750-11-103)
Supplement: Additional file 1 — Total Dry Biomass in Various Carbon Sources. Biomass was harvested from strains grown on carbon sources as shown for 12, 24 and 36 hours at 30°C. The strains are QM 6a, JKTR2-6, and the cre1 deletion strain. Dry weight is shown in mg per ml. [file 1472-6750-11-103-S1.DOC]

**Additional File 1.**

**Supplementary Table 1**

|  | **12 hours** | | | **24 hours** | | | **36 hours** | | |
| --- | --- | --- | --- | --- | --- | --- | --- | --- | --- |
| **Carbon Source** | **QM6a** | ***cre2-*** | ***cre1-*** | **QM6a** | ***cre2-*** | ***cre1-*** | **QM6a** | ***cre2-*** | ***cre1-*** |
| 2.0% Glucose | 3.37 ± 0.41 | 2.72 ± 0.1 | 1.21 ± 0.10 | 5.19 ± 0.29 | 4.40 ± 0.25 | 2.25 ± 0.06 | 6.00 ± 0.45 | 4.97 ± 0.06 | 3.57 ± 0.12 |
| 2.0% Maltose | 0.52 ± 0.07 | 0.85 ± 0.02 | 0.32 ± 0.05 | 1.20 ± 0.26 | 3.64 ± 0.07 | 0.26 ± 0.02 | 3.89 ± 0.83 | 5.32 ± 0.41 | 0.26 ± 0.10 |
| 2.0% Lactose | 0.56 ± 0.03 | 0.53 ± 0.04 | 0.57 ± 0.05 | 1.05 ± 0.08 | 0.62 ± 0.12 | 0.81 ± 0.05 | 2.29 ± 0.11 | 1.27 ± 0.08 | 1.55 ± 0.04 |
| 2.0% Glycerol | 0.84 ± 0.11 | 0.85 ± 0.08 | 0.73 ± 0.10 | 2.09 ± 0.32 | 2.06 ± 0.08 | 1.96 ± 0.05 | 3.71 ± 0.28 | 3.21 ± 0.08 | 3.44 ± 0.17 |
| 2.0% Sorbitol | 0.55 ± 0.06 | 0.54 ± 0.06 | 0.32 ± 0.07 | 0.89 ± 0.08 | 0.94 ± 0.02 | 0.31 ± 0.06 | 1.48 ± 0.05 | 1.50 ± 0.12 | 0.51 ± 0.06 |
