# Supplementary figures and images for: Disruption of Trichoderma reesei cre2, encoding an ubiquitin C-terminal hydrolase, results in increased cellulase activity
Source: BMC Biotechnol. 2011 Nov 9;11:103. doi: 10.1186/1472-6750-11-103 (PMC3226525; doi:10.1186/1472-6750-11-103)

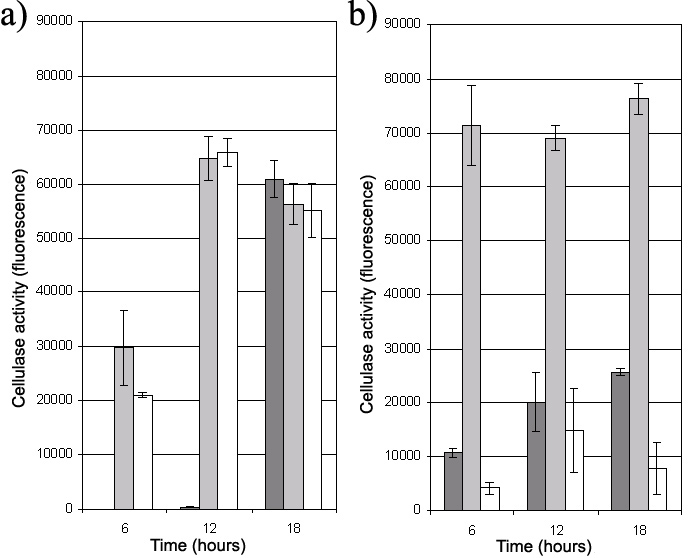

Supplement: Additional file 2 — Total secreted cellulase activity of three T. reesei strains. Cellulase secretion of T. reesei strains QM 6a (dark grey shading), JKTR2-6 (light grey shading) and cre1 deletion (white shading) measured using the EnzChek Cellulase Substrate. Mycelia for inoculation were harvested after growth for 28 hours in 1% (w/v) glucose medium. Mycelia were washed with liquid carbon free medium and 50 mg was added to 5 ml culture medium in 10 ml culture bottles. Cultures were grown at 30°C, shaken at 200 RPM, and were harvested 6 hours, 12 hours and 18 hours post inoculation. Time indicated as hours post transfer. Error bars indicate standard deviations of duplicate cultures each analysed in duplicate. (a) Repressing conditions, growth in medium containing 1% glucose and 1 mM sophorose. (b) Derepressing conditions, growth in medium containing 1% sorbitol and 1 mM sophorose. When diluted, the measured cellulase activity of these samples decreased proportionally, showing the detection limit of the assay had not been exceeded. [file 1472-6750-11-103-S2.DOC]
