# Supplementary material for: Disruption of Trichoderma reesei cre2, encoding an ubiquitin C-terminal hydrolase, results in increased cellulase activity
Source: BMC Biotechnol. 2011 Nov 9;11:103. doi: 10.1186/1472-6750-11-103 (PMC3226525; doi:10.1186/1472-6750-11-103)
Supplement: Additional file 3 — Secreted Cellulase and Xylanase Activities. Cellulase (a) or Xylanase (b) secretion in T. reesei strains QM 6a, JKTR2-6, and the cre1 disruption strain measured using the EnzChek Cellulase Substrate (a) or the EnzChek Xylanase Assay Kit (b) uncorrected for dry weight. Time indicated as hours post transfer, grown at 30°C. Error margins indicated standard deviations determined from the result of assay duplicates and biological triplicates. [file 1472-6750-11-103-S3.PDF]

Additional File 3.

Supplementary Table 2

a. Total Cellulase activity

	12 hours			24 hours			36 hours		
**Carbon Source**	**QM6a**	***cre2-***	***cre1-***	**QM6a**	***cre2-***	***cre1-***	**QM6a**	***cre2-***	***cre1-***
2.0% Glucose	0	0	80 ± 10	0	0	6050 ± 1400	0	0	29200 ± 2100
2.0% Lactose	540 ± 180	900 ± 220	1480 ± 500	14300 ± 1130	21100 ± 1300	9180 ± 630	23900 ± 1130	25600 ± 600	24000 ± 800
2.0% Glucose / 2.0% Lactose	0	0	5900 ± 500	0	0	32300 ± 1800	0	470 ± 200	62500 ± 2500
2.0% Sorbitol	0	50 ± 10	130 ± 40	0	440 ± 130	190 ± 40	0	700 ± 80	780 ± 50
2.0% Glycerol	110 ± 340	1570 ± 370	5090 ± 840	240 ± 80	750 ± 130	2170 ± 1140	0	100 ± 50	0

b Total Xylanase activity

	12 hours			24 hours			36 hours		
**Carbon Source**	**QM6a**	***cre2-***	***cre1-***	**QM6a**	***cre2-***	***cre1-***	**QM6a**	***cre2-***	***cre1-***
2.0% Glucose	0	0	34 ± 10	0	0	1160 ± 290	0	0	5840 ± 440
2.0% Lactose	540 ± 180	900 ± 210	1490 ± 500	14280 ± 1130	21080 ± 1300	9180 ± 630	23880 ± 1130	25630 ± 600	23960 ± 790
2.0% Glucose / 2.0% Lactose	0	0	1130 ± 120	0	0	9810 ± 980	0	50 ± 90	17610 ± 1980
