# Supplementary material for: Disruption of Trichoderma reesei cre2, encoding an ubiquitin C-terminal hydrolase, results in increased cellulase activity
Source: BMC Biotechnol. 2011 Nov 9;11:103. doi: 10.1186/1472-6750-11-103 (PMC3226525; doi:10.1186/1472-6750-11-103)
Supplement: Additional file 4 — Dry weights for Avicel and Carboxymethyl Cellulose cultures. Data for Figure 6. Conidia were added to 100 ml culture medium in 250 ml Erlenmeyer flasks. Cultures were grown at 30°C, shaken at 1500 RPM, for 24 hours, in triplicate. The carbon sources are 1% glucose plus 0.5% avicel, 0.1% fructose plus 0.5% avicel, 1% glucose plus 0.5% carboxymethyl cellulose, 0.1% fructose plus 0.5% carboxymethyl cellulose. [file 1472-6750-11-103-S4.DOC]

**Additional File 4.**

**Supplementary Table 3**

| Strain | Condition | Weight gm |
| --- | --- | --- |
| QM6a | Avicel | 0.22 |
| QM6a | Avicel + G | 0.57 |
| *cre2-* | Avicel | 0.20 |
| cre2- | Avicel + G | 0.51 |
| QM6a | CMC | 0.31 |
| QM6a | CMC + G | 0.49 |
| cre2- | CMC | 0.18 |
| cre2- | CMC + G | 0.28 |
